# Impact of fluorescent protein fusions on the bacterial flagellar motor

**DOI:** 10.1038/s41598-017-11241-w

**Published:** 2017-10-03

**Authors:** M. Heo, A. L. Nord, D. Chamousset, E. van Rijn, H. J. E. Beaumont, F. Pedaci

**Affiliations:** 1 Single-molecule Biophysics dept, Centre de Biochimie Stucturale, CNRS UMR5048 UM INSERM U1054, 29 Rue de Navacelles, 34090 Montpellier, France; 2 Department of Bionanoscience, Kavli Institute of Nanoscience, Delft University of Technology, 2628 CJ Delft, The Netherlands

## Abstract

Fluorescent fusion proteins open a direct and unique window onto protein function. However, they also introduce the risk of perturbation of the function of the native protein. Successful applications of fluorescent fusions therefore rely on a careful assessment and minimization of the side effects, but such insight is still lacking for many applications. This is particularly relevant in the study of the internal dynamics of motor proteins, where both the chemical and mechanical reaction coordinates can be affected. Fluorescent proteins fused to the *stator* of the Bacterial Flagellar Motor (BFM) have previously been used to unveil the motor subunit dynamics. Here we report the effects on single motors of three fluorescent proteins fused to the stators, all of which altered BFM behavior. The torque generated by individual stators was reduced while their stoichiometry remained unaffected. MotB fusions decreased the switching frequency and induced a novel bias-dependent asymmetry in the speed in the two directions. These effects could be mitigated by inserting a linker at the fusion point. These findings provide a quantitative account of the effects of fluorescent fusions to the stator on BFM dynamics and their alleviation— new insights that advance the use of fluorescent fusions to probe the dynamics of protein complexes.

## Introduction

Fusion of a fluorescent protein to a protein of interest is an invaluable tool for the study of protein function in a broad, and still expanding range of *in vivo* and *in vitro* systems. However, it is widely recognized that the presence of fluorescent proteins can alter functional properties of the native protein^1–5^. Careful assessment of these side effects and strategies to minimize them have therefore been critical to the success of fluorescent protein fusions (FPFs)^6–9^. The continuing development of FPF-based approaches to explore novel phenomena also calls for new insight into the associated side effects and methods to alleviate them^10–12^. One direction of research for which this holds is the study of the internal dynamics of proteins complexes^13,14^.

Recently, several studies have made use of FPFs to study the internal subunit dynamics of the bacterial flagellar motor (BFM)^15–29^. Although FPFs were shown to affect BFM function by decreasing the chemotactic motility of cells and average speed of motors^15^, a quantitative characterization of the impact on the BFM mechanical behavior is lacking. Such perturbations limit the depth to which the internal dynamics and function of the BFM, and protein complexes in general, can be probed with FPFs. Linker peptides inserted at the fusion point have been instrumental in minimizing non-native behaviour of FPFs in many biological systems^8–11^, but their use in the study of protein-complex dynamics is limited in the BFM has thus far been limited.

**Figure 1:**
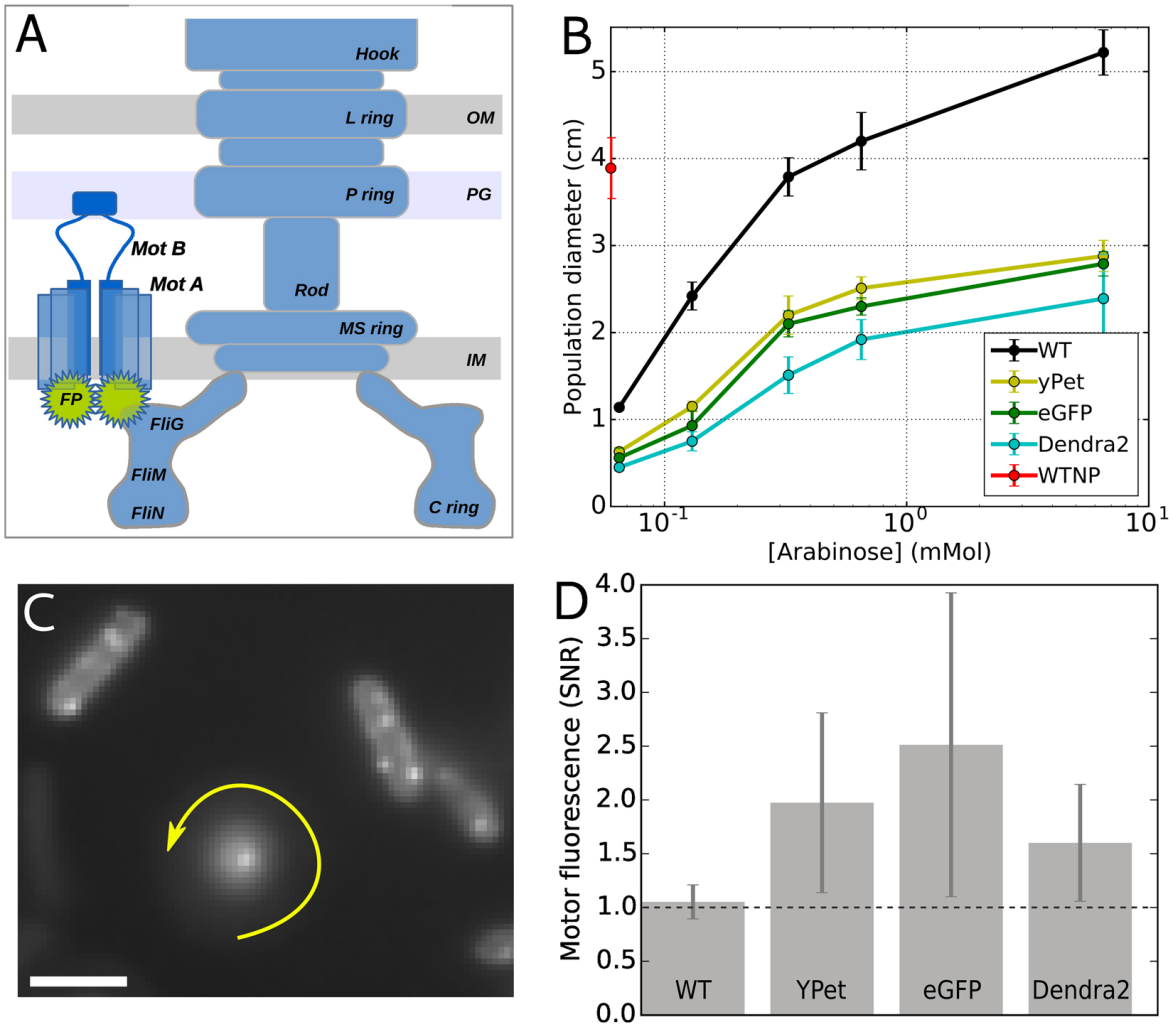
(**A**) Schematic structure of the BFM with one stator labeled by a fluorescent protein fused on the N-terminus of MotB (OM: Outer membrane, PG: peptidoglygan, IM: internal membrane). (**B**) Population chemotactic motility on soft agar plates. The diameter of the ring formed by motile populations after 8 h is shown as a function of stator induction, showing a decreased chemotactic motility in strains with labeled stators (error bars indicate standard deviation, number of trials: 3). Red point provides a comparison of the mean and standard deviation of WT with the native promoter, performed in the absence of arabinose. (**C**) Stator fluorescence detection from an active labeled motor. In the center, a tethered cell rotates around the bright spot corresponding to the tagged BFM (eGFP-MotB here). The image is the sum of several frames, therefore blurring indicates the cell rotation. In each frame, the rotating cell is visible with different orientations. Fluorescently tagged motors are also visible as bright spots in cells stuck on the glass surface. Scale bar: 2 *μ*m. (**D**) Signal to noise ratio (SNR) for motor fluorescence detected exclusively in rotating tethered cells as in C ([Arabinose] = 0.13 mM). Absence of fluorescence is reflected by the line SNR = 1 (see Supplementary Fig. S2 for further information). Error bars indicate standard deviation (number of motors measured: 5, 12, 15, and 16 for WT, YPet, eGFP, and Dendra2, respectively).

The BFM is located at the base of each flagellum in the membrane of many motile bacteria (Fig. 1A). The torque generated by the complex, and the consequent rotation of the flagella, powers chemotactic cellular motility along chemical gradients^30–32^. Chemotaxis is achieved by chemostimulus-controlled modulation of the motor rotational bias. When all motors of the cell spin counterclockwise (CCW), the cell swims in a straight ‘run’, whereas the switch of one or more motors to the clockwise (CW) direction causes the cell to ‘tumble’^33^. The CCW to CW bias of a single motor is regulated by the intracellular concentration of the response regulator of the chemotaxis-signaling pathway, CheY-P^34–36^. Torque is generated by up to a dozen stator units, which use the cellular ion motive force (IMF) to perform work on the rotor part of the BFM^31^. In the bacterium *Escherichia coli*, each stator is an ion channel composed of four MotA subunits and two MotB subunits. The stators dynamically turn over between two populations: one that is bound to the BFM and one that passively diffuses in the inner membrane^15,37^. These dynamics have been observed directly at the single-stator level using fluorescent fusions to the N-terminus of MotB^15,18,19,21,28,29^. More recently, similar fusions have been used to unveil that the number of stators recruited into the complex depend on the load of the BFM^18,19,29^.

In large protein complexes such as the BFM and, for example, polymerases or ribosomes, function emerges from a myriad of dynamical interactions between the subunits. While FPFs have proven to be a very powerful tool to study these complexes, it is likely that they impart biologically relevant functional perturbations. Here, aiming to address this, we fused different fluorescent proteins to the N-terminus of MotB and characterized the effects at the level of individual BFMs. The results show that the observed decrease in population-level chemotactic motility is underpinned by changes, some of which unexpected, in key mechanical parameters of the BFM rotation. On the other hand, stator stoichiometry results were unaffected by the presence of the label. Furthermore, we show that both choosing the right fluorescent protein and introducing a rigid linker at the fusion point can mitigate all of these side-effects.

## Results

### Functional stators, but reduced chemotactic motility

After fusing the fluorescent proteins YPet, eGFP and Dendra2 directly to the N-terminus of MotB (FPs-MotB), as done in several studies with (e)GFP^15,18,19,21,29,38^, we verified that the constructed strains were motile in soft agar at the population level (Fig. 1B). Previous studies using eGFP-MotB used a construct containing 500 bp upstream of and including the first 28 codons of *motB* (encompassing the putative membrane-targeting sequence), followed by *eGFP* and then the first 500 bp of *motB* ^15,18,21^. We compared this construct to one in which eGFP was fused directly to the N-terminus of MotB and found that the latter performed similarly or slightly better in tests of chemotactic population motility (Supplementary Fig. S1).

At the two induction levels tested, the chemotactic population motility of cells expressing FPs-MotB was reduced by ~50% relative to cells expressing wild-type MotB, in line with what was previously found for a GFP-MotB fusion^15,38^. When observed in TIRF, tethered cells were often found to rotate around a fixed bright spot, corresponding to the location of the rotating BFM (one example is shown in Fig. 1C). Image analysis of the fluorescence signal of functional motors in rotating tethered cells, taking into account the auto-fluorescence of the cells and the background signal level, shows that the motor signal-to-noise ratio is higher than one in FPs-MotB strains, while remains equal to one in WT (see Fig. 1D and Supplementary Fig. S2). Together, these results indicate that the motors are functional, that the labeled stators are localized at the motor, and that all fluorescent proteins fused to MotB impacted BFM function.

### Symmetric speed reduction and speed asymmetry in tagged motors

**Figure 2:**
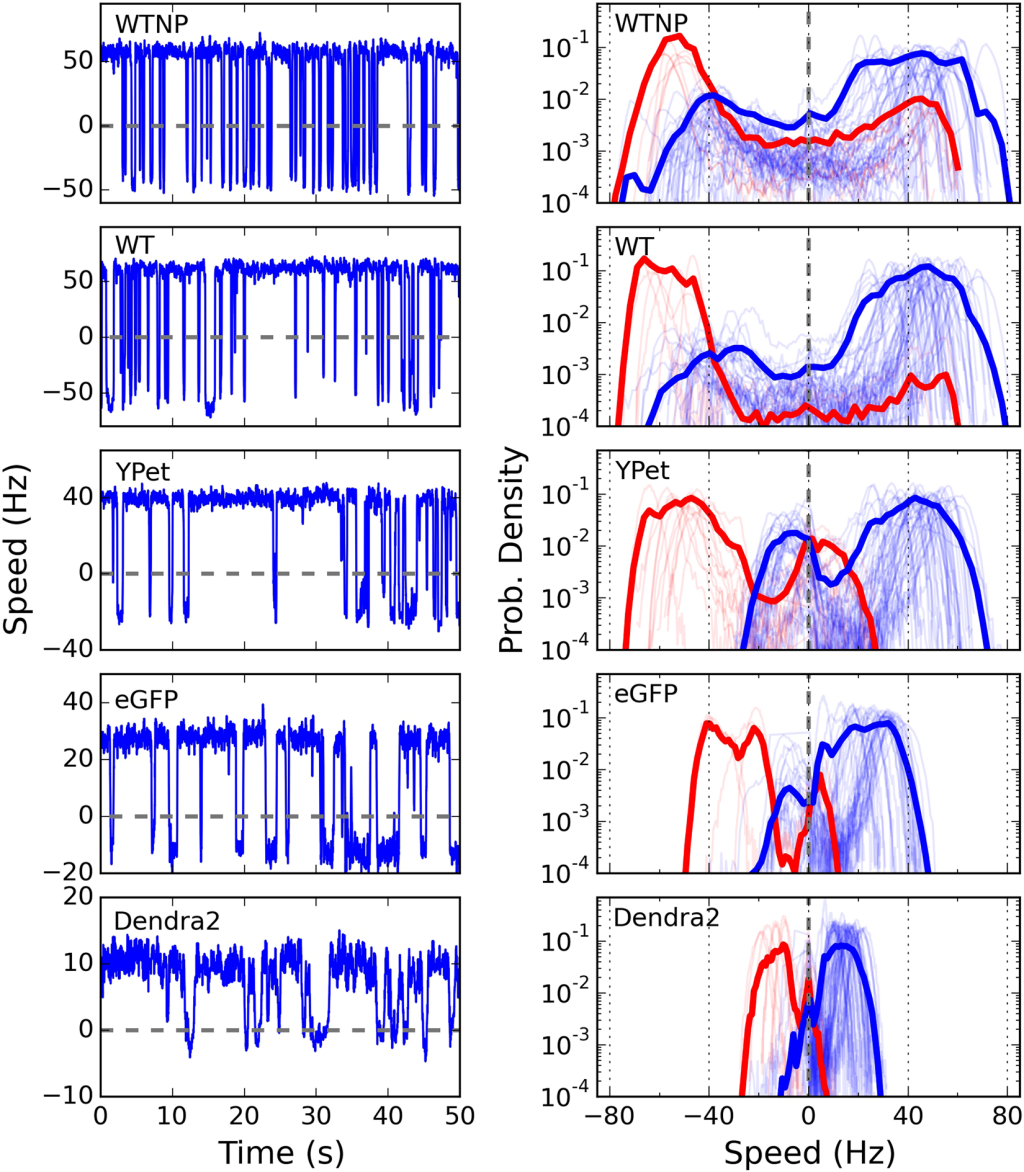
Left column: example speed traces of individual CCW-biased motors rotating 1.1 *μ*m beads. Right column: probability distributions of the speed. Positive and negative speeds indicate CCW and CW rotation direction, respectively. Blue and red indicate CCW and CW biased motors, respectively. Individual thin lines show individual motor measurements, and the thick lines show the average of all measurements (number of motors measured: 51, 69, 57, 42, and 43 for WTNP, WT, YPet, eGFP, and Dendra2, respectively). The induction level of stators in WT, YPet, eGFP and Dendra2 is set by an arabinose concentration of 6.5 mM (see Supplementary Fig. S3 for the same measurements at a lower induction level). WTNP: WT with native promoter.

A reduction in chemotactic population motility on soft agar for the FP-MotB strains can be the result of different factors, such as decreased BFM torque, a suboptimal switching frequency (tumbling allows cells to escape dead-ends in the agar matrix^39^), a suboptimal response of the BFM to CheY-P concentrations (i.e. the output of the chemotaxis signaling pathway), or changes in other BFM dynamics. To get insight into the effect of the fluorescent tag on the motor, we used a tethered-bead assay to measure the rotational speed of individual motors. Figure 2 shows the speed distributions for single motors, from all the strains, rotating a high load (1.1 *μ*m diameter bead). The speed distributions are colored according to their bias, with blue and red indicating CCW (counter clockwise, positive speed) and CW (clockwise, negative speed) biased motors, respectively. WT (wild type stators, inducible plasmid) and WTNP (wild type stators, native promoter, see Table 1) behave similarly: regardless of their bias, they reach nearly the same absolute value of speed (±50 Hz) in both directions, in line with previous results^40^. In BFMs with wild-type stators, this behavior is not affected by a change in the induction level of MotB (Supplementary Fig. S3).

**Table 1:** *E*. *coli* strains used in this work.

Ref.Name	Strain	Genome	Plasmid	Assay
WTNP	MT02	*FliC* ^st^	—	tethered-bead
FP-MotB	JPA605	*FliC* ^st^, Δ*MotAB*	*MotA* FP-*MotB*	tethered-bead, tethered-cell
WT	JPA604	Δ*MotAB*	*MotA MotB*	chemotactic motility
FP-MotB	JHC36^59^	*FliC* ^st^, Δ*MotAB*, Δ*CheY*	*MotA* FP-*MotB*	tethered-bead torque steps

All strains come from the parent strain RP437^60^. FliC^st^ indicates the hydrophobic variant of FliC (producing “sticky” filaments^61^). The fluorescent proteins (FP) fused to MotB are YPet, eGFP, and Dendra2. All plasmids were pBAD33 vector, carrying chloramphenicol resistance and induced by L-arabinose. Strains JPA604, JPA605, and MT02 were gifts from R.M. Berry lab.

However, a tagged motor behaves differently. First, regardless of the bias, the most visited speeds of the FP-MotB strains in the two directions are lower than in WT and WTNP. The amount of decrease depends on the tag: YPet reaches almost WT speeds, while eGFP can reach only ~±30 Hz, and Dendra2 only ~±20 Hz. This *symmetric reduction* of the most visited speed affects motors biased in both directions. Second, a novel feature arises when one considers the bias of the tagged motors. Contrary to WT, when a tagged motor switches from its preferred direction of rotation, it reaches only a fraction of its previous speed in the opposite direction. In FP-MotB strains, this is evidenced in the global average distributions (Fig. 2) by the secondary blue and red peaks at negative and positive speeds, respectively. We label this effect *speed asymmetry*. It is observed in a given strain for both biases: tagged CCW-biased motors switch from a high CCW speed to a lower CW speed, while CW-biased motors switch from a high CW speed to a lower CCW speed. The speed asymmetry of tagged motors is therefore bias-dependent, and always results in a lower speed in the less visited direction of rotation.

**Figure 3:**
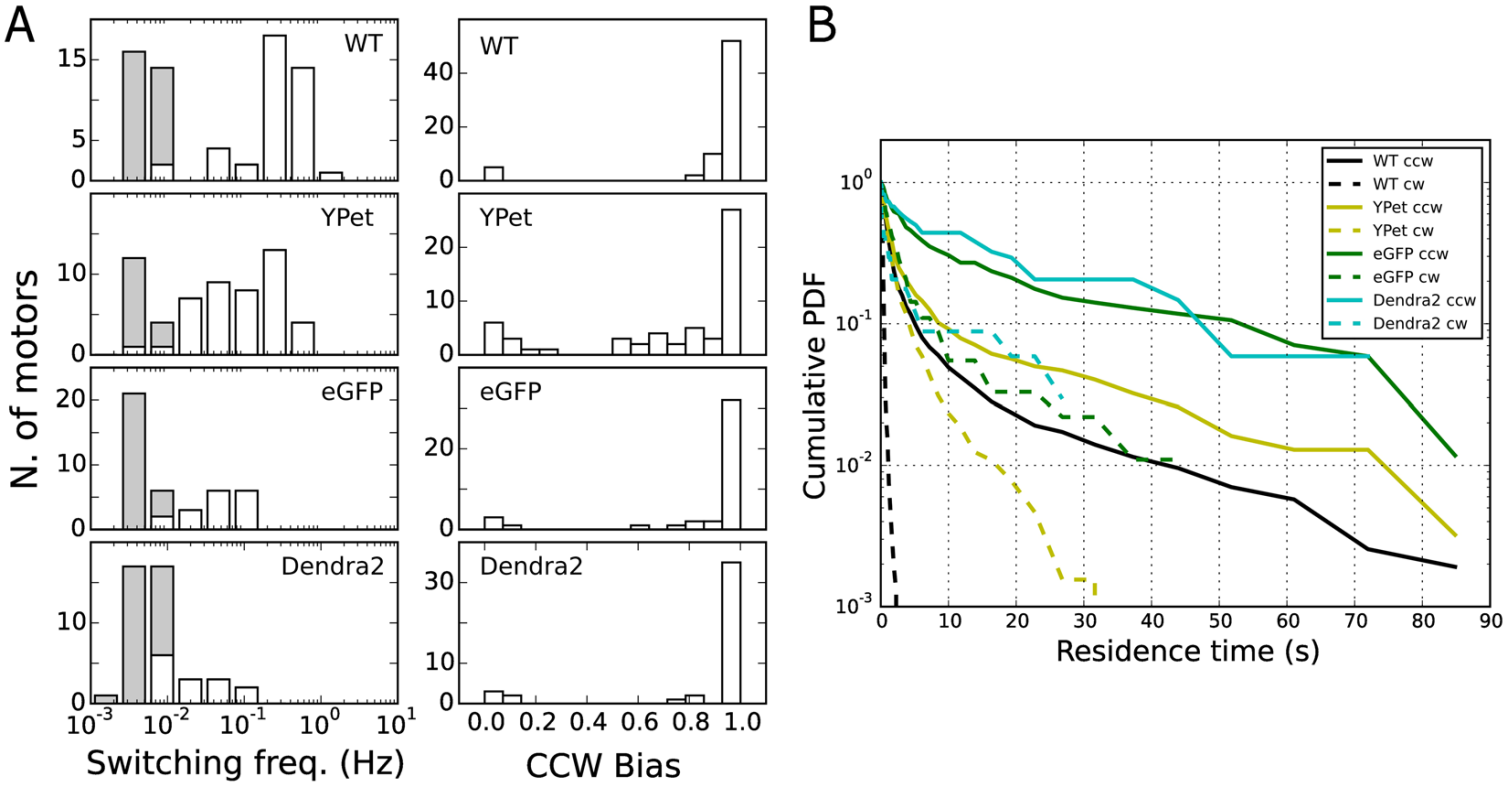
(**A**) Distributions of switching frequency (left column) and CCW bias (right column) for all the strains tested. Gray columns indicate motors which did not switch during the measurement (their switching frequency has been set equal to twice the inverse of the measurement time, and it should be considered as an upper limit). (**B**) Reverse cumulative probability distribution of the time spent in CW and CCW (indicating at time *t*_*o*_ the percentage of observed residence times longer than *t*_*o*_^43^) for all (CCW biased) motors of the different strains (number of motors measured: 69, 57, 42, and 43 for WT, YPet, eGFP, and Dendra2, respectively). Stator induction is set by a concentration of arabinose of 6.5 mM (see Supplementary Figs S3–S4 for the same measurements at a lower induction).

It has previously been shown that the bias of an individual motor, that is, the percentage of time the motor spends rotating CCW, shows a sigmoidal relationship with the concentration of CheY-phosphate (CheY-P) within the cell^35^. CheY-P is the output of the chemotactic signal transduction network which detects changes in the chemical composition of the environment^41^. This sigmoidal relationship, characterized by a large Hill coefficient, leads to a bimodal distribution of motor bias^42^, as seen in Fig. 3A. For WT, we measure a switching frequency and distribution of motor bias that is in line with previous results^35,42^. However, in the tagged motors, we observe a reduction of the frequency of switches with respect to WT (Fig. 3A, left panels), with a severity that depends on the particular FP and reflects the order observed above for the decrease in speed (from the least to the most affected: YPet, eGFP, Dendra2). The distribution of motor biases, on the other hand, is less affected (Fig. 3A right panels). The reverse cumulative distribution of the residence times (indicating at time *t*_*o*_ the percentage of observed residence times longer than *t*_*o*_^43^) is shown in Fig. 3 for the CCW and CW states. As observed previously, we find that the residence times are distributed non-exponentially with long tails, potentially due to the presence of signaling noise within the chemotaxis network^43^. Reflecting their decreased switching frequency, tagged motors show extended residence times in both directions of rotation with respect to WT.

### Torque and stoichiometry of tagged stators

**Figure 4:**
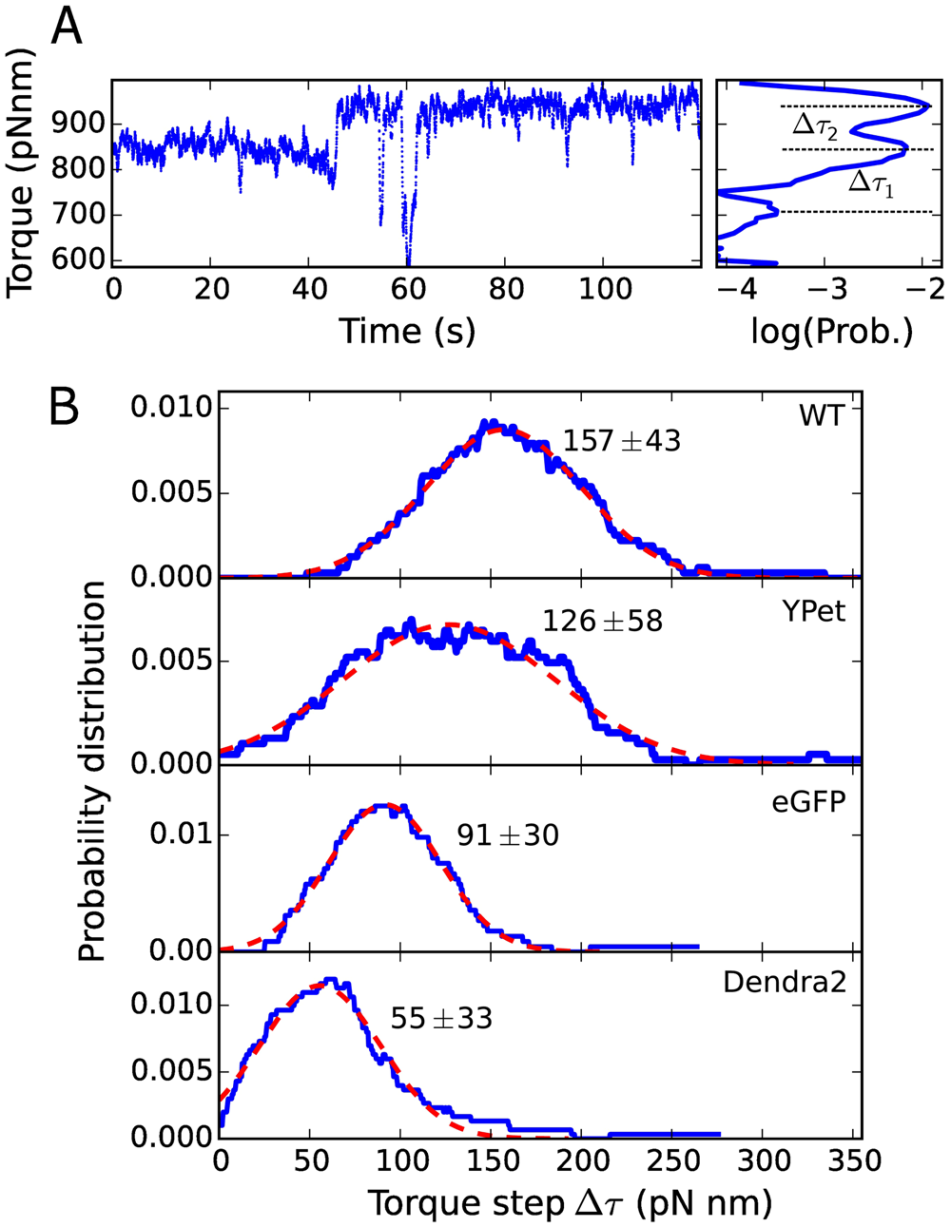
(**A**) An example torque time trace (left) in WT, showing spontaneous stator turnover as steps in torque level. The separation between neighboring torque levels (Δ*τ*_*i*_) is determined from a multiple Gaussian fit of the torque histogram (right). (**B**) Distributions of the distances between neighboring Gaussians (Δ*τ*_*i*_) in the multiple Gaussian fit of motor torque, measured in single torque traces like A (number of motors measured: 17, 21, 23, and 29 for WT, YPet, eGFP, and Dendra2, respectively). The peak indicates the average torque produced by the exchange of a single stator. A single Gaussian fit (red dashed line) provides the center and standard deviation of the peak (indicated by the text in each panel). Welch’s t-test applied to each pair of distributions rejects the null hypothesis in all cases (p-value threshold of 0.05). Stator induction is set by a concentration of arabinose of 6.5 mM.

Decreased populaton-level chemotactic motility and an overall decrease of speed in tagged motors could be due to either a lower torque generated by individual stators or by a lower number of stators bound to the BFM, relative to WT. To discriminate between these two possibilities, we have analyzed the discrete steps in the torque traces which occur spontaneously due to stator turnover^15^ in single motors. For this analysis, we used strains lacking the switching regulating protein CheY to avoid the complication of switching events. Figure 4A shows an example torque trace from WT at steady-state, where different torque levels due to stator turnover are detected, and Fig. 4B shows the distribution of the torque contributed by a single stator for all the recordings (see Methods and Materials). Gaussian fits to the distributions, reflecting the average torque contribution of a single stator, give the values of 157 ± 43 pN nm in WT, 126 ± 58 pN nm in YPet, 91 ± 30 pN nm in eGFP, and 55 ± 33 pN nm in Dendra2 fusions. Welch’s t-test applied to each pair of single-stator torque distributions shows that these values are statistically different (Methods and Materials). Thus, the torque generated by a single stator decreases in the tagged motors, following the same trend observed for the symmetric decrease of speed (Fig. 2) and for the decrease in switching frequency (Fig. 3).

Moreover, the ratio between the torque per stator in tagged motors and in WT measured here reflects closely the ratio of the most visited speeds (either CW or CCW) in tagged motors and in WT, shown in Fig. 2. This implies that the number of stators does not change significantly in tagged motors with respect to WT or WTNP, and that the symmetric speed reduction observed in the tagged motors is mainly the result of the reduced torque generated by each stator. Considering the torque produced by *N* stators as *Nτ*_1_ = *γ*2*πω*_*N*_ (where *τ*_1_ is the torque produced by a single stator, *γ* the drag coefficient of the bead, and *ω*_*N*_ the measured speed (in Hz) of the motor with *N* stators), all the strains tested are driven by *N* ~ 8–10 stators, in line with previous measurements at high load^15,44^. This result supports previous works focused on quantifying steady-state stator stoichiometry by fluorescent stators^15,18–21,28,29,45–47^.

### A linker improves the performance of tagged stators

Aiming to mitigate the effects described above in tagged motors, we introduced a linker between the N-terminus of MotB and the fluorophore. We have tested two types of linkers, one rigid (EAAAK) and one more flexible (GGGGS)^11^, both in one copy or as a triple repeat. Population motility measurements (shown in Supplementary Fig. S6) show the results of these tests in Dendra2. Generally, we found that a rigid and longer linker demonstrated a greater improvement in motor performance that a flexible and shorter linker, respectively, with a linker composed of a triple EAAAK repeat showing the greatest improvement.

**Figure 5:**
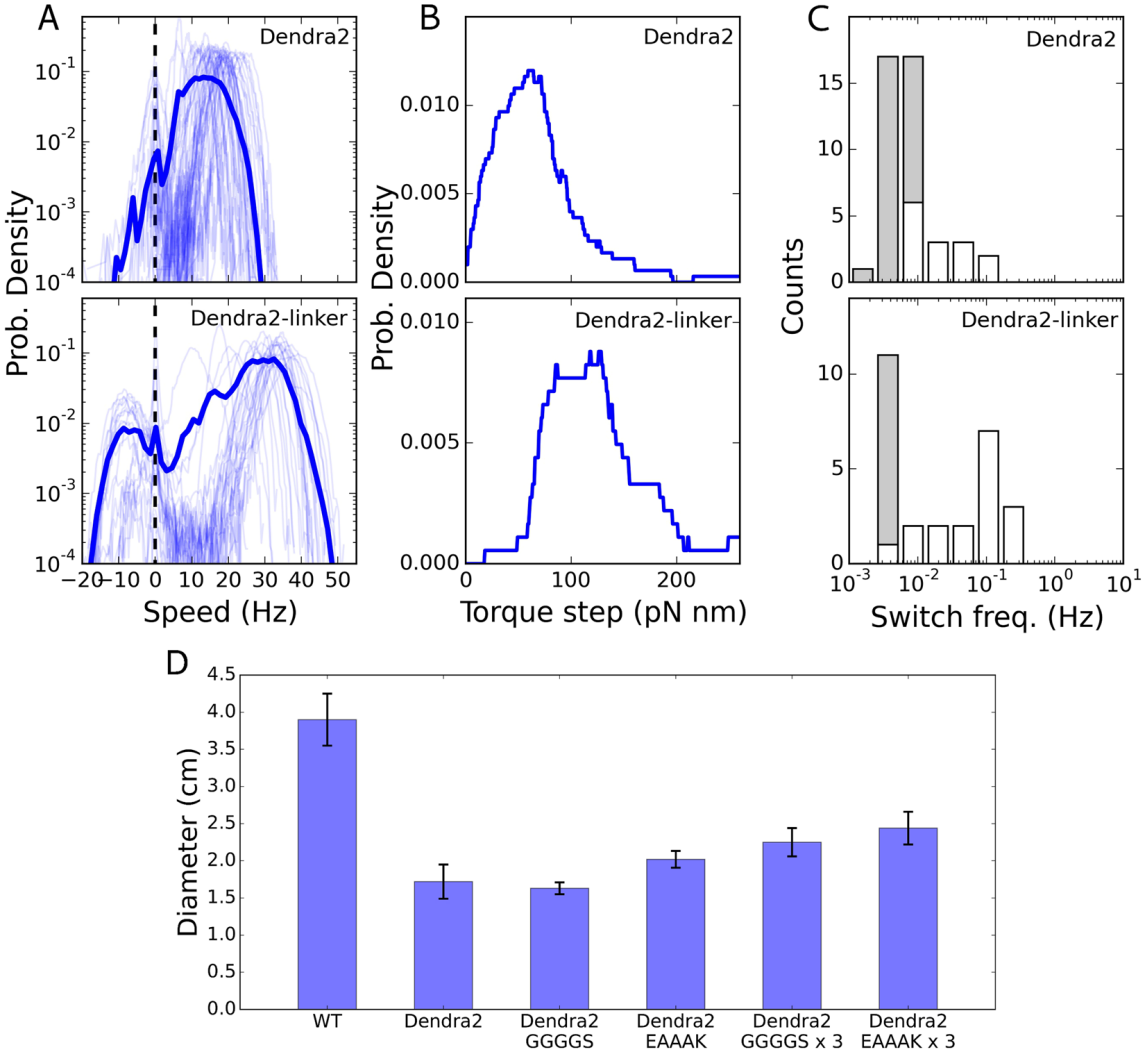
Introducing a linker between the FP and MotB improves the features of the tagged motors. (**A**–**C**) We show here the results for Dendra2-MotB (and in Supplementary Fig. S5 we show the analysis for YPet-MotB and eGFP-MotB). Top (low) panels correspond to the absence (presence) of the (EAAAK)_3_ linker. (**A**) Probability distribution of the speed (as in Fig. 2) of CCW biased Dendra2-MotB motors (CW biased motors are not shown due to low statistics). (**B**) Probability distribution of the torque steps observed at steady-state during stator turnover (as in Fig. 4B). (**C**) Distribution of measured switching frequency (as in Fig. 3A). Number of motors measured in (**A**–**C**): 54 and 30 for the direct fusion and the linker strain, respectively). [Arabinose] = 6.5 mM. (**D**) Chemotaxis motility comparisons of the Dendra2 fusion stators. Error bars give standard deviation over three measurements. The rigid linker (EAAAK) yields a larger improvement than the flexible (GGGGS) linker, and the triple repeat yields a larger improvement than the single.

Focusing on the Dendra2 fusion, which most affects the dynamics of the BFM, in Fig. 5 we compare single Dendra2 motors in the presence and absence of the (EAAAK)_3_ linker (here for CCW biased motors only, due to low statistics for CW bias). In the presence of the linker, the most visited CCW speed increases up to ~30 Hz (Fig. 5A), while it is only ~18 Hz in the absence of the linker (and ~50 Hz in WT, see Fig. 2). Therefore, the overall speed reduction relative to WT is mitigated by the presence of the linker. We have seen above (Fig. 2) that switching in Dendra2 motors is actually more similar to a pause, as the least visited speed remains peaked at zero. In the presence of the linker, motor switching is restored, though a degree of speed asymmetry remains, and the CW rotation is recuperated with a speed peaked at ~−10 Hz. Figure 5B shows that the higher speed in the presence of the linker is paralleled by a higher torque generated by each stator (with ~100 pN nm per stator in the presence of the linker, ~55 pN nm in Dendra2 without the linker, and ~160 pN nm in WT, see Fig. 4). As before, this suggests that the number of stators in the motor is similar in the presence and absence of the linker (*N* ~ 8–9), and that the decrease in overall speed is due to a lower torque (and speed) generated by each stator. Finally, Fig. 5C shows the change in switching frequency, which increases in the presence of the linker and moves towards that of WT (as shown in Fig. 3A). Similar to Dendra2, in YPet and eGFP tagged motors the presence of the linker is responsible for a partial recovery of speed and decrease in severity of the speed asymmetry, as shown in Supplementary Fig. S7.

## Discussion

Fluorescent protein fusions have been successfully used to study the internal dynamics of protein complexes, but insight into the effect of the label on biologically relevant functions of the complex is still limited. We examined this for fluorescent fusions similar to those that have previously been used to shed light on the internal dynamics of the BFM^15,18,19,21,28,29^. Globally, we find that the fusion of an FP at the N-terminus of MotB affects several features in the dynamical behavior of the motor driving a high load. The overall speed of the BFM is reduced due to a reduction of the torque produced by each stator, while the stator occupancy remains unaffected. Also, the switching frequency of tagged motors decreases, both the CCW and CW residence times increase, while the global distribution of motor bias is less affected. Finally, the fusions to MotB induce a novel BFM dynamic that involves an asymmetry in the speeds attained in opposing rotational directions, depending on the motor’s bias. These effects of the fluorescent fusion proteins on the BFM could be partially restored by a rigid linker (EAAAK)_3_ inserted at the fusion point. Together, these findings reveal how a fluorescent protein fusion that does not abolish function completely can modulate biologically relevant dynamics of a protein complex and even induce new behavior, all of which can be partially relieved by the incorporation of a linker.

The fact that an FP fused to the stator causes an overall decrease of speed could be explained by a variety of mechanisms. For example, the tags could perturb ion translocation, hinder the stator conformational changes involved in force generation, inhibit the interaction between the stator and rotor, introduce an extra drag in the rotor, or interfere with stator recruitment. While our measurements cannot discriminate a single mechanism, our analysis shows that the number of stators is not affected in tagged motors, ruling out an interference with stator recruitment, and confirming that the effects we observe are induced by the physical presence of the labels on the stators in the motor.

The fusion proteins also lowered the switching frequency. It has previously been observed that the switching frequency is dependent upon motor torque and rotation speed^48^, and it has been proposed that the conformation of the switch complex is dependent upon the interactions with the stators in a torque-dependent manner^49,50^. It is thus plausible that the observed decrease in switch frequency is due to a decrease in the single stator torque due to the FP fusion. Alternatively, it could be that the fusion protein causes a reduced switching frequency by direct interactions with the governing structural elements.

The unexpected discovery that FP-MotB fusions cause a bias-dependent speed asymmetry indicates that the fluorescent protein interacts with a yet unspecified asymmetry in the stator-rotor interaction that may or may not be present in the native BFM. Importantly, we note that the mechanisms suggested above affect speed symmetrically in both directions, and cannot account alone for the observations of speed asymmetry. Further study is needed to resolve the exact mechanism that gives rise to the observed bias-dependent speed asymmetry. In the Supplementary Material we speculate about possible mechanisms that could be responsible for this asymmetry.

It is increasingly clear that the selection of a proper linker is an important element of FPF design^10,11^ While properties such as linker flexibility, length, and hydrophobicity are important, predicting the success of a linker *a priori* is difficult. Here, a longer linker performed better than a shorter linker, and a rigid linker performed better than a flexible linker. We hypothesize that these particular properties allow for a sufficient spatial separation between MotB and the FP, thereby reducing the interactions between the FP and the force-generating MotB/FliG interface.

Sequence alignment of the three FPs (see SI) show them to be least similar at the N and C terminal regions, both of which are exposed on the protein surface where they may interact with MotA and MotB. It is plausible that structural differences in these regions may explain the different behavior that we observe for the different MotB fusions. Whether the order of impact of the FPs observed here is universal or specific to MotB remains to be discovered. Depending on the environment and concentration, many FPs are prone to dimerization and oligimerization, presenting another factor for consideration.

In science, the act of observing often impinges upon the phenomenon of interest. The power of fluorescent protein fusions as a tool for the study of protein function hinges on our understanding of their side effects. Here we have thoroughly characterized these effects for the stator of the BFM. The findings advance our ability to study flagellar protein-complex dynamics while imparting minimal perturbation.

## Methods

### Bacteria and culture preparation

All the *E*. *coli* strains used are detailed in Table 1. Three FPs (eGFP, YPet, and Dendra2) were fused either directly to the N-terminus of MotB or with a linker in between, as detailed below. A cassette containing the *motA* and FP-fused *motB* genes was cloned downstream of the promotor in plasmid pBAD33 (ara, araC, pACYC184/p15A)^51^ and transformed into the cells. Cells were grown at 33^o^C in tryptone broth with chloramphenicol (34 μg/ml) and either 0.13 mM or 6.5 mM of L-arabinose to an OD^600^ of 0.55–0.65. For all of the individual cell measurements, the flagella were mechanically sheared^52^ before the cells were centrifuged and resuspended in motility buffer (10 mM potassium phosphate, 0.1 mM EDTA, 10 mM lactic acid, pH 7.0). Measurements were carried out at 20^o^C in a simple flow chamber made by two coverslips separated by a layer of parafilm. The population-level motility was measured by observing the chemotactic population front on soft (0.25%) agar plates. As cells metabolize nutrients in the agar, they create an attractant gradient and, if chemotactic, swim out from the point of inoculation, forming an expanding ring whose diameter was measured after 8 h (each measurement was repeated in triplicate, see Fig. 1B). In order to choose the best linker, the population motility was measured for Dendra2 stators joined to four different linkers of the following sequences (see Fig. 5D): (GGGGS), (EAAAK), (GGGGS)_3_, and (EAAAK)_3_.

### Motor rotation measurements

Speed measurements of individual motors were performed using a tethered-bead assay^52^, as follows. Bacterial cells were immobilized to poly-L-Lysine (Sigma) coated coverslips, and polystyrene beads (1.1 *μ*m diameter, Sigma) were allowed to spontaneously attach to truncated ‘sticky’ (FliC^st^) filaments. Motor rotation was measured by tracking the rotation of the bead, which was monitored with a bright-field laser microscope setup^53^. Images of the bead hologram were recorded by a CMOS camera (Optronics) at the rate of 300 or 500 frames per second for 3 to 4 minutes. Where tested, the linker joining MotB to the FP was (EAAAK)_3_.

Custom Labview and Python software was used to track the bead with nm resolution, correct for sample drift, and fit an ellipse to the trajectory (considering the ellipse as the projection of a tilted circle). The angle of the bead with respect to the ellipse determined the angular position, and the angular speed of motor rotation was calculated from the time derivative of the angular position. The speed was then median-filtered with a window of 70 ms. Positive (negative) speed indicates CCW (CW) rotation. From the speed trace, motor bias was measured as the proportion of time spent rotating CCW. A motor that spent more than 50% of the time rotating in the CCW (CW) direction is referred to as CCW- (CW-) biased. Speed histograms of individual motors were constructed with a sub-Hz bin-width. The individual normalized histograms are shown as light lines in Fig. 2 as probability densities (in logarithmic scale). The global average distributions are shown by the thick lines. The line color of the speed distributions in Fig. 2 reflects the motor bias, blue and red indicating CCW- and CW-biased motors, respectively. Rotational switching events were detected from the median-filtered speed trace, based on an algorithm which finds the crossing of two thresholds, set at 2/3 of the mean speed in each direction^54^. The switching frequency of a motor was calculated by the number of detected switching events divided by the duration of the measurement.

### Estimate of single stator torque contribution

The torque generated by the motor was calculated as the product of the drag coefficient of the bead^55^ and the speed. In several motors at steady-state (here ΔCheY to avoid complications due to switching) we could observe jumps between discrete torque levels (one example is shown in Fig. 4A). For each of these traces, the histogram of motor torque had multiple peaks, which we fit with a multiple Gaussian fit. Under the common assumption that discrete changes in motor torque are due to a change in stator number^21,37,44,52,56,57^, expected due to stator-turnover^15^, the distance in the histogram from one peak to the next represents the torque contributed by a single stator. The distance between neighboring Gaussians was calculated for each individual trace, and the distribution of such single-stator torque contributions for all the measured motors for WT and all of the FP-MotB strains is shown in Fig. 4B. Finally, a Gaussian was fit to this distribution to determine the average single-stator torque contribution. A Welch’s unequal variances t-test was applied between each of the single-stator torque distributions to test the null hypothesis that any two population means are equal (using a two-tailed test). The threshold applied to the p-values was 0.05.

### Fluorescence microscopy measurements

Fluorescence measurements of individual motors were performed using a tethered-cell assay^58^, as follows. Bacterial cells were allowed to spontaneously adhere to the glass coverslip via the truncated hydrophobic filament. Cells which tethered by a single filament rotated around the axis of the corresponding flagellar motor (Fig. 1C).

Fluorescence excitation was performed at the wavelength of 488 nm in a TIRF configuration (at a power of ~200 W/cm^2^, measured before the objective) and emission was detected by an EMCCD camera (Andor) in the range of 500–550 nm. The emission of Dendra2, a photo-switchable fluorophore, was detected in its unconverted form. Tethered cells with labeled motors were often observed rotating around a bright fluorescent spot, indicating the location of the functional motor. The fluorescence intensity of nine pixels around the center of rotation was summed to obtain the motor signal *S*_*m*_. Only fluorescent spots which coincided with the center of rotation of a rotating cell were analyzed, to avoid false positives due to e.g. possible clusters of FPs. Due to rotation and blur, the auto-fluorescence of a rotating cell was not possible to quantify reliably; therefore, the auto-fluorescence of ~20 stuck cells in the same field of view was averaged to obtain the noise level *S*_*n*_. Figure 1D shows the signal-to-noise ratio, defined by SNR = *S*_*m*_/*S*_*n*_, for the different strains. An SNR of one indicates that the motor is no more fluorescent than the autofluorescent cellular background. An SNR greater than one indicates the presence of fluorophores at the motor, indicating proper folding of the fluorescent protein and successful integration of the stator to the motor.

## Electronic supplementary material

SI

## References

[1] Costantini LM, Snapp EL (2015). Going viral with fluorescent proteins. Journal of virology.

[2] Gahlmann A, Moerner WE (2014). Exploring bacterial cell biology with single-molecule tracking and super-resolution imaging. Nat Rev Micro.

[3] Baens M (2006). The dark side of egfp: Defective polyubiquitination. PLOS ONE.

[4] Margolin W (2012). Price of tags in protein localization studies. Journal of Bacteriology.

[5] Swulius MT, Jensen GJ (2012). Helical mreb cytoskeleton in escherichia coli mc1000/ple7 is an artifact of the n-terminal yellow fluorescent protein tag. Journal of Bacteriology.

[6] Snapp, E. *Current Protocols in Cell Biology*, chap. Design and Use of Fluorescent Fusion Proteins in Cell Biology (John Wiley and Sons, Inc., 2005). 10.1002/0471143030.cb2104s27 PMC2875081 18228466

[7] Snapp EL (2009). Fluorescent proteins: a cell biologist’s user guide. Trends in Cell Biology.

[8] Huang L, Pike D, Sleat DE, Nanda V, Lobel P (2014). Potential pitfalls and solutions for use of fluorescent fusion proteins to study the lysosome. PLOS ONE.

[9] Crivat G, Taraska JW (2012). Imaging proteins inside cells with fluorescent tags. Trends in biotechnology.

[10] Arai R, Ueda H, Kitayama A, Kamiya N, Nagamune T (2001). Design of the linkers which effectively separate domains of a bifunctional fusion protein. Protein Engineering, Design and Selection.

[11] Chen X, Zaro JL, Shen W-C (2013). Fusion protein linkers: property, design and functionality. Advanced drug delivery reviews.

[12] Giraldez T, Hughes TE, Sigworth FJ (2005). Generation of functional fluorescent bk channels by random insertion of gfp variants. The Journal of general physiology.

[13] Norris SR, Núñez MF, Verhey KJ (2015). Influence of fluorescent tag on the motility properties of kinesin-1 in single-molecule assays. Biophys. J..

[14] Agbulut, O. *et al*. GFP expression in muscle cells impairs actin-myosin interactions: implications for cell therapy. *Nature Methods* (2006). 10.1038/nmeth0506-331 16628201

[15] Leake MC (2006). Stoichiometry and turnover in single, functioning membrane protein complexes. Nature.

[16] Delalez NJ (2010). Signal-dependent turnover of the bacterial flagellar switch protein FliM. PNAS.

[17] Delalez NJ, Berry RM, Armitage JP (2014). Stoichiometry and turnover of the bacterial flagellar switch protein FliN. MBio.

[18] Tipping MJ, Delalez NJ, Lim R, Berry RM, Armitage JP (2013). Load-dependent assembly of the bacterial flagellar motor. MBio.

[19] Lele PP, Hosu BG, Berg HC (2013). Dynamics of mechanosensing in the bacterial flagellar motor. PNAS.

[20] Paulick A (2015). Dual stator dynamics in the Shewanella oneidensis MR-1 flagellar motor. Mol. Microbiol..

[21] Tipping MJ, Steel BC, Delalez NJ, Berry RM, Armitage JP (2013). Quantification of flagellar motor stator dynamics through *in vivo* proton-motive force control. Mol. Microbiol..

[22] Hosu BG, Nathan VSJ, Berg HC (2016). Internal and external components of the bacterial flagellar motor rotate as a unit. Proceedings of the National Academy of Sciences.

[23] Branch RW, Sayegh MN, Shen C, Nathan VS, Berg HC (2014). Adaptive remodelling by flin in the bacterial rotary motor. Journal of Molecular Biology.

[24] Lele PP, Berg HC (2015). Switching of bacterial flagellar motors [corrected] triggered by mutant flig. Biophysical journal.

[25] Lele PP, Branch RW, Nathan VSJ, Berg HC (2012). Mechanism for adaptive remodeling of the bacterial flagellar switch. PNAS.

[26] Yuan J, Branch RW, Hosu BG, Berg HC (2012). Adaptation at the output of the chemotaxis signalling pathway. Nature.

[27] Fukuoka H, Inoue Y, Terasawa S, Takahashi H, Ishijima A (2010). Exchange of rotor components in functioning bacterial flagellar motor. Biochem Biophys Res Commun.

[28] Morimoto YV, Nakamura S, Hiraoka KD, Namba K, Minamino T (2013). Distinct roles of highly conserved charged residues at the mota-flig interface in bacterial flagellar motor rotation. J. Bacteriol..

[29] Che Y-S (2014). Load-sensitive coupling of proton translocation and torque generation in the bacterial flagellar motor. Mol. Microbiol..

[30] Berg HC (2003). The rotary motor of bacterial flagella. Annu. Rev. Biochem..

[31] Sowa Y, Berry RM (2008). Bacterial flagellar motor. Q. Rev. Biophys..

[32] Morimoto YV, Minamino T (2014). Structure and function of the bi-directional bacterial flagellar motor. Biomolecules.

[33] Turner L, Ryu WS, Berg HC (2000). Real-time imaging of fluorescent flagellar filaments. Journal of bacteriology.

[34] Welch, M., Oosawa, K., Aizawa, S. & Eisenbach, M. Phosphorylation-dependent binding of a signal molecule to the flagellar switch of bacteria. *Proc Natl Acad Sci USA* **90** (1993). 10.1073/pnas.90.19.8787 PMC47445 8415608

[35] Cluzel P, Surette M, Leibler S (2000). An ultrasensitive bacterial motor revealed by monitoring signaling proteins in single cells. Science.

[36] Sourjik V, Berg HC (2002). Binding of the escherichia coli response regulator chey to its target measured *in vivo* by fluorescence resonance energy transfer. Proc Natl Acad Sci USA.

[37] Block, S, M. & Berg, H. Successive incorporation of force-generating units in the bacterial rotary motor. *Nature* 470–472 (1984). 10.1038/309470a0 6374467

[38] Morimoto YV, Nakamura S, Kami-ike N, Namba K, Minamino T (2010). Charged residues in the cytoplasmic loop of MotA are required for stator assembly into the bacterial flagellar motor. Mol. Microbiol..

[39] Bubendorfer S, Koltai M, Rossmann F, Sourjik V, Thormann KM (2014). Secondary bacterial flagellar system improves bacterial spreading by increasing the directional persistence of swimming. Proceedings of the National Academy of Sciences of the United States of America.

[40] Yuan J, Fahrner KA, Turner L, Berg HC (2010). Asymmetry in the clockwise and counterclockwise rotation of the bacterial flagellar motor. PNAS.

[41] Eisenbach M (1996). Control of bacterial chemotaxis. Mol Microbiol.

[42] Montrone M, Eisenbach M, Oesterhelt D, Marwan W (1998). Regulation of switching frequency and bias of the bacterial flagellar motor by chey and fumarate. J Bacteriol.

[43] Park H, Oikonomou P, Guet CC, Cluzel P (2011). Noise underlies switching behavior of the bacterial flagellum. Biophys. J..

[44] Reid SW (2006). The maximum number of torque-generating units in the flagellar motor of escherichia coli is at least 11. PNAS.

[45] Zhu S (2014). Conformational change in the periplamic region of the flagellar stator coupled with the assembly around the rotor. PNAS.

[46] Brenzinger, S. *et al*. Mutations targeting the plug-domain of the *Shewanella Oneidensis* proton-driven stator allow swimming at increased viscosity and under anaerobic conditions. *Mol*. *Microbiol*. **105** (2016). 10.1111/mmi.13499 27611183

[47] Fukuoka H, Yakushi T, Kusumoto A, Homma M (2005). Assembly of motor proteins, PomA and PomB, in the Na+ driven stator of the flagellar motor. J. Mol. Biol..

[48] Fahrner KA, Ryu WS, Berg HC (2003). Biomechanics: bacterial flagellar switching under load. Nature.

[49] van Albada SB, Tanase-Nicola S, ten Wolde PR (2009). The switching dynamics of the bacterial flagellar motor. Molecular Systems Biology.

[50] Bai F, Minamino T, Wu Z, Namba K, Xing J (2012). Coupling between switching regulation and torque generation in bacterial flagellar motor. Phys. Rev. Lett..

[51] Guzman LM, Belin D, Carson MJ, Beckwith J (1995). Tight regulation, modulation, and high-level expression by vectors containing the arabinose pbad promoter. Journal of bacteriology.

[52] Ryu WS, Berry RM, Berg HC (2000). Torque-generating units of the flagellar motor of escherichia coli have a high duty ratio. Nature.

[53] Dulin D, Barland S, Hachair X, Pedaci F (2014). Efficient illumination for microsecond tracking microscopy. PloS One.

[54] Bai F (2010). Conformational spread as a mechanism for cooperativity in the bacterial flagellar switch. Science.

[55] Leach J (2009). Comparison of faxén’s correction for a microsphere translating or rotating near a surface. Phys. Rev. E.

[56] Blair DF, Berg HC (1988). Restoration of torque in defective flagellar motors. Science.

[57] Inoue Y (2008). Torque-speed relationships of na+-driven chimeric flagellar motors in escherichia coli. J Mol Biol.

[58] Silverman M, Simon M (1974). Flagellar rotation and the mechanism of bacterial motility. Nature.

[59] Sowa Y, Homma M, Ishijima A, Berry RM (2014). Hybrid-fuel bacterial flagellar motors in escherichia coli. Proceedings of the National Academy of Sciences of the United States of America.

[60] Parkinson JS (1978). Complementation analysis and deletion mapping of escherichia coli mutants defective in chemotaxis. Journal of bacteriology.

[61] Scharf BE, Fahrner KA, Turner L, Berg HC (1998). Control of direction of flagellar rotation in bacterial chemotaxis. PNAS.

