# Supplementary material for: Impact of fluorescent protein fusions on the bacterial flagellar motor
Source: Sci Rep. 2017 Oct 3;7:12583. doi: 10.1038/s41598-017-11241-w (PMC5626733; doi:10.1038/s41598-017-11241-w)
Supplement: Supplementary file 1 — SI [file 41598_2017_11241_MOESM1_ESM.pdf]

Supplementary Information.

Impact of fluorescent protein fusions on the bacterial flagellar motor

M Heo[1+], AL Nord[1+], D Chamousset[1], E van Rijn[2], HJE Beaumont[2], F Pedaci[1*]

1 Single-molecule Biophysics dept, Centre de Biochimie Stucturale, CNRS UMR5048 UM INSERM U1054, 29 Rue de Navacelles, 34090 Montpellier, France.

2 Department of Bionanoscience, Kavli Institute of Nanoscience, Delft University of Technology, 2628 CJ, Delft, The Netherlands.

* francesco.pedaci@cbs.cnrs.fr

+ these authors contributed equally to this work.

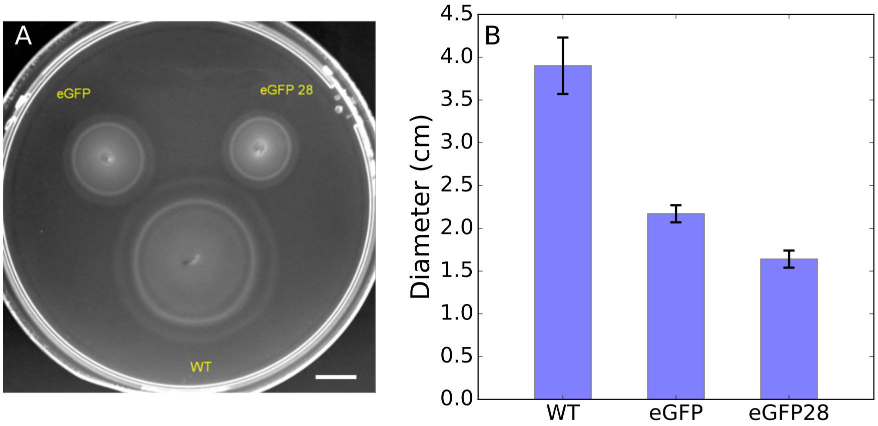

Figure S1: A comparison of a direct fusion of eGFP and a previous construct containing 500bp upstream of and including the first 28 codons of *motB* (encompassing the putative membrane-targeting sequence), followed by *eGFP* and then the first 500bp of *motB* [1–3]. A) A photograph of a single test of population-level motility on a soft (0.25%) agar plate. Scale bar is 1 cm. B) Comparison of the chemotaxis diameter measured after eight hours for wild-type (WT) and the to two eGFP constructs. Error bars show standard deviation over 5 trials similar to that shown in (A).

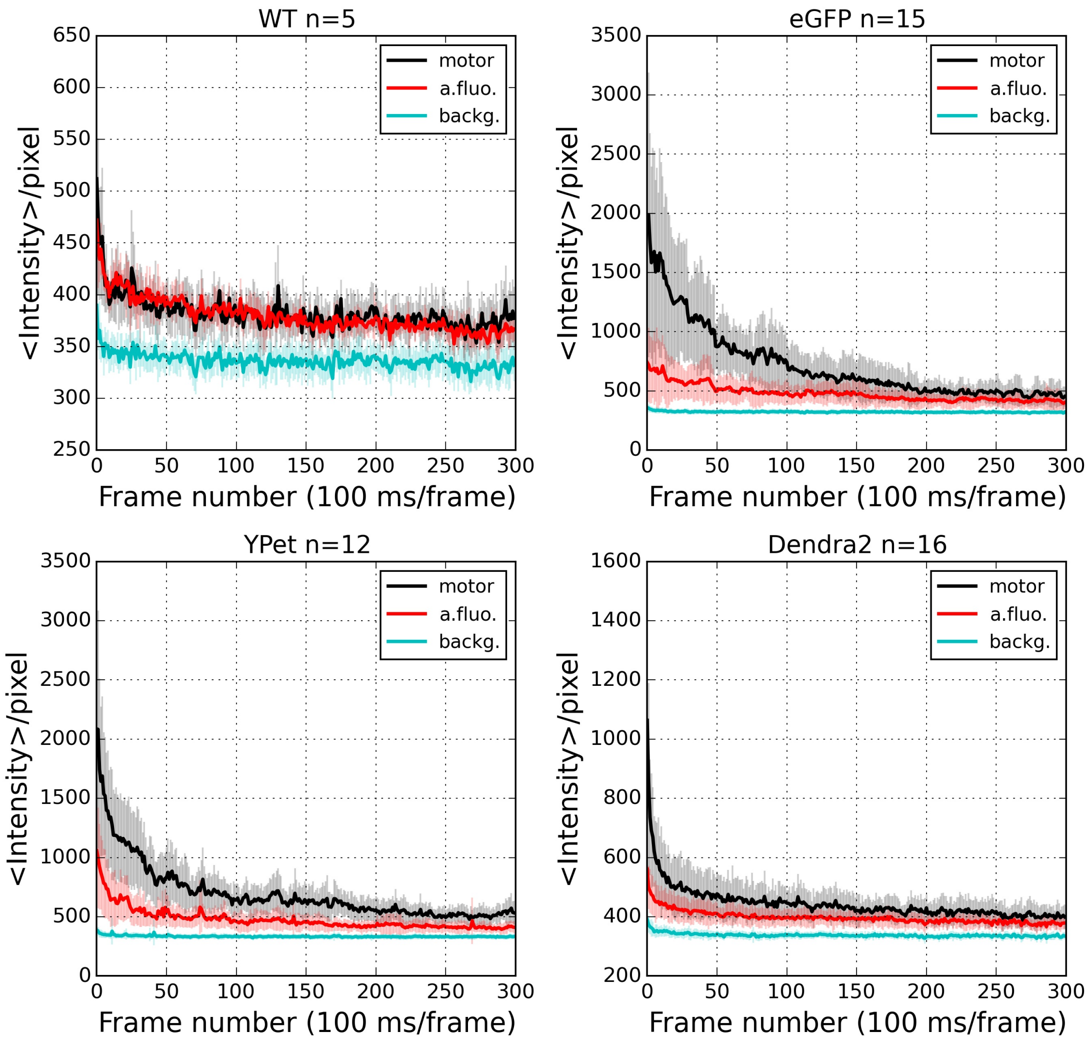

Figure S2: Fluorescence time traces. For each strain we have measured the time evolution of the fluorescence signal from a 3x3 pixels region around an active motor (black line) on n rotating tethered cells (the strain and n are indicated in the title of each panel). The cellular auto-fluorescence signal (red) is measured on 3x3 pixel regions selected over several (~ 20) stuck cells in the same field of view. The background signal (cyan) is measured on regions not occupied by cells. [Arabinose] $= 0.13$ mM. Error bars indicate the standard deviation.

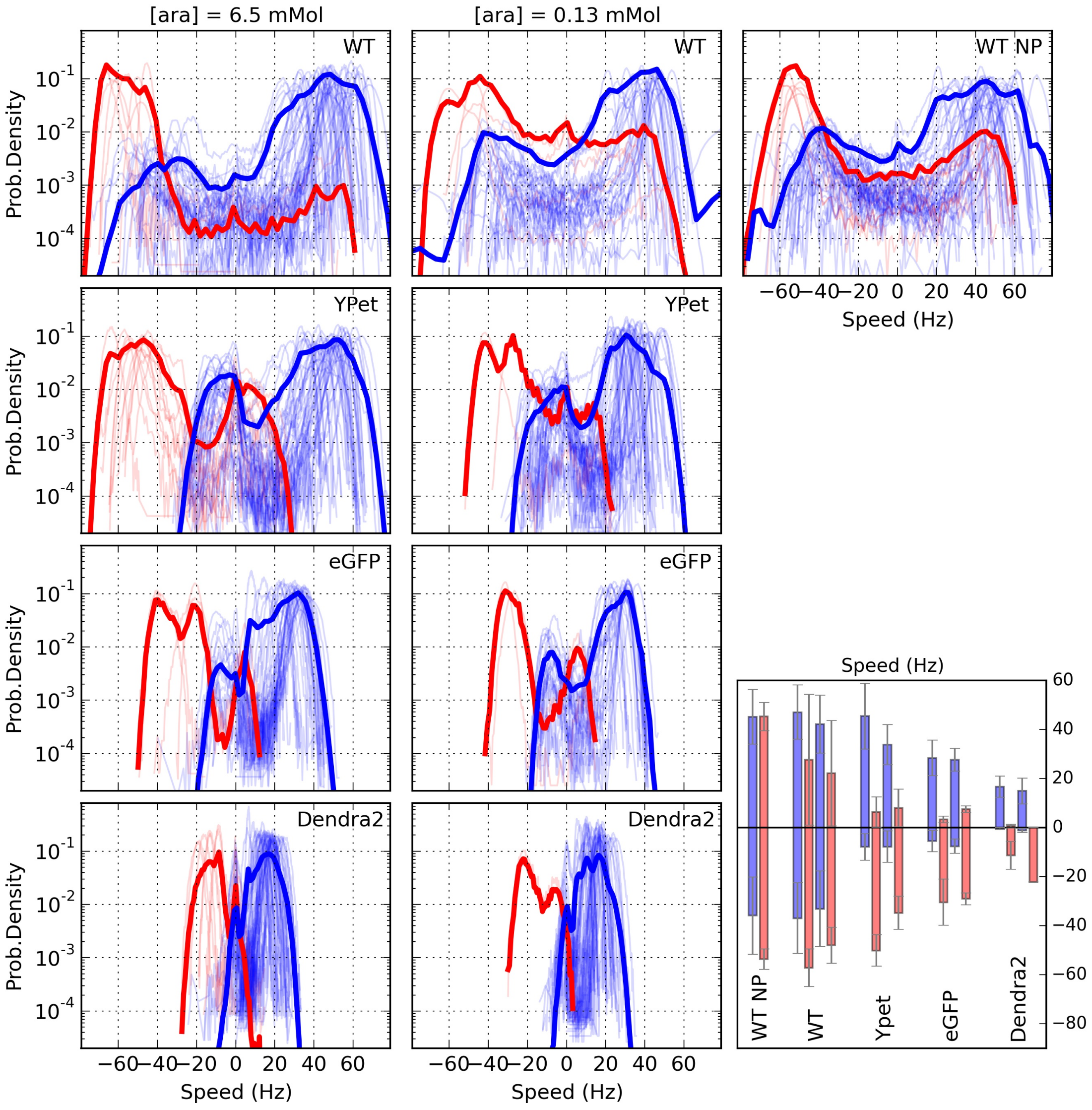

Figure S3: Speed histograms for the two induction levels used (first column: [Ara] = 6.5 mM, second column: [Ara] = 0.13 mM, while WTNP corresponds to the native expression). As in Fig.2 of the main text, positive (negative) speed indicates CCW (CW) direction, and blue (red) indicates CCW (CW) biased motors. Individual thin lines show individual motor measurements, and the thick lines show the average of all measurements. The bar plot at the bottom right summarizes the mean speed and standard deviation for each strain for both expression levels, for CCW and CW biased motors (blue and red bars, respectively). For the strains with the stators on a plasmid, WT, YPet, eGFP, and Dendra2, the first two bars correspond to high induction (6.5 mM), the second two bars to low induction (0.13 mM). Number of motors measured: 69, 57, 42, and 43 at 6.5 mM [Ara] and 29, 39, 24, and 19 at 0.13 mM [Ara] for WT, YPet, eGFP, and Dendra2, respectively, and 51 for WTNP.

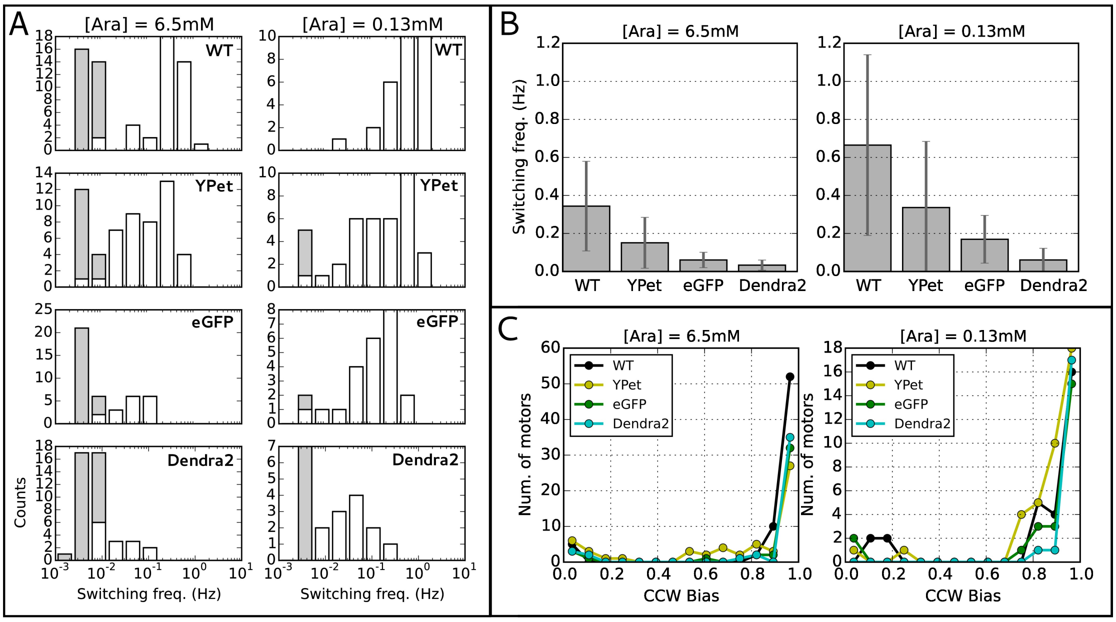

Figure S4: A) Distributions of switching frequency for all the strains, at the two induction levels tested (indicated in the title of the two columns). As in Fig.3A, the gray bars indicate motors which did not switch during the measurement, so their switching frequency has been set equal to twice the inverse of the measurement time, and it should be considered as an upper limit. B) Summary of switching frequency mean and standard deviation for all the strains at the two induction levels tested. C) Distributions of the CCW bias for all the strains at the two induction levels. Number of motors measured: 69, 57, 42, and 43 at 6.5 mM [Ara] and 29, 39, 24, and 19 at 0.13 mM [Ara] for WT, YPet, eGFP, and Dendra2, respectively.

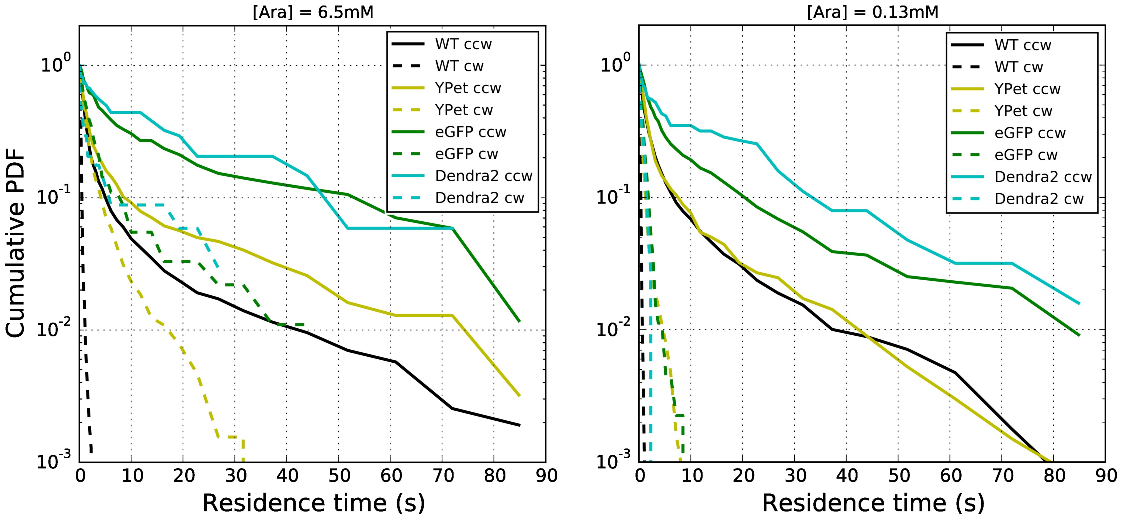

Figure S5: Inverse cumulative distribution (as in Fig.3B) of the residence times for all CCW biased motors at the two induction levels tested, indicated in the figure titles. Number of motors measured: 69, 57, 42, and 43 at 6.5 mM [Ara] and 29, 39, 24, and 19 at 0.13 mM [Ara] for WT, YPet, eGFP, and Dendra2, respectively.

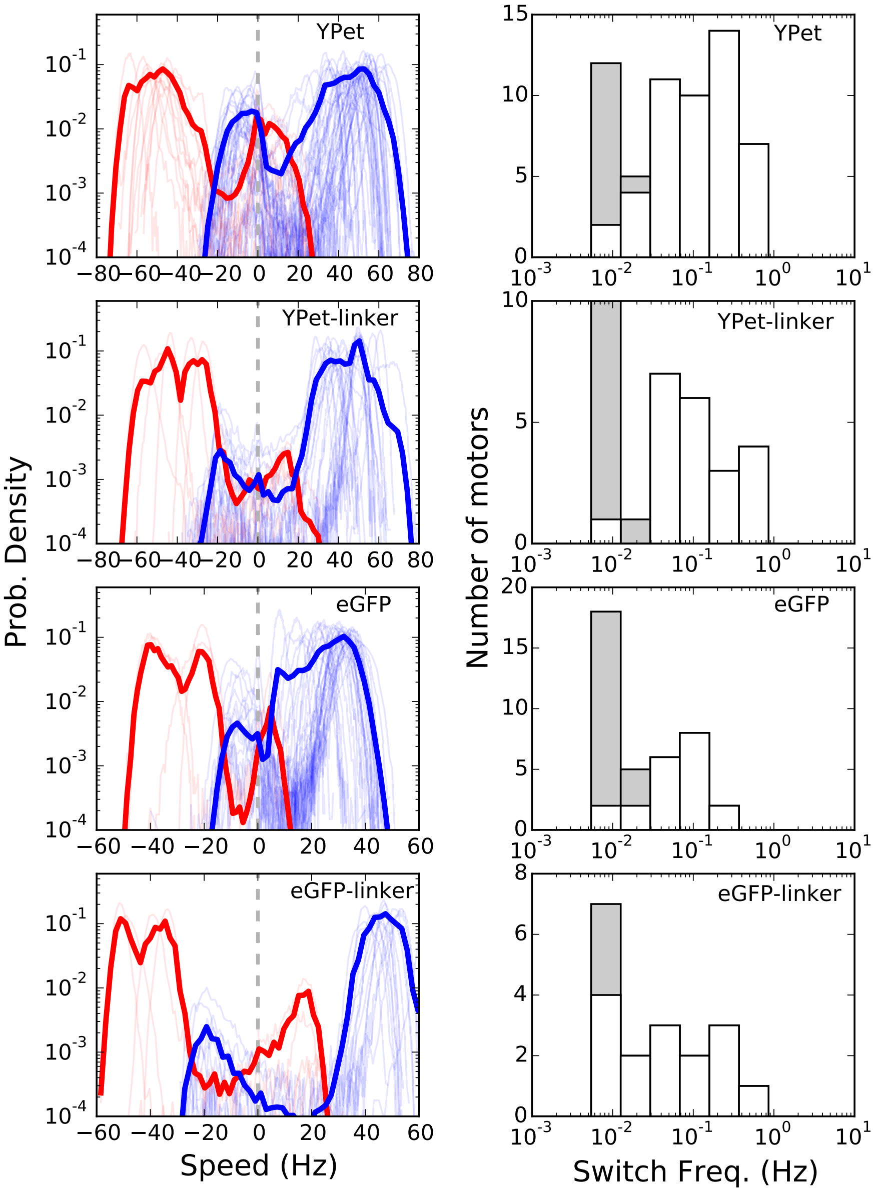

Figure S6: The $(EAAAK)_3$ linker improves the performance of YPet and eGFP motors. Left column: Distributions of the speed measured in YPet and eGFP motors, in the presence and absence of the linker, as indicated in each panel (the data in the absence of linkers are the same as in Fig.2, shown as a reference). Right column: distributions of the switching frequency measured for the same strains. As in Fig.3 and Fig. S4, the gray bars indicate motors which did not switch during the measurement, so their switching frequency should be considered as an upper limit. Number of motors measured: 47, 37, 30, and 18 for YPet, YPet with a linker, eGFP, and eGFP with a linker, respectively. [Ara] = 6.5 mM

Possible origins of the speed asymmetry in switching

One first hypothetical mechanism that could explain the asymmetry in switching can be an asymmetric effect of the tags on the power stroke of the stator. Following a recent model proposing that the two pairs of the cytoplasmic loops of MotA may drive the rotation in opposite directions [4], one could speculate that the presence of two tags on MotB could perturb one pair of the cytoplasmic MotA loops more severely than the other, decreasing the efficiency of torque generation in one direction more than in the other. In this scenario, different stators should then assemble with the same orientation of their efficient MotA pairs with respect to FliG, otherwise a random proportion of efficient and inefficient MotA pairs of different stators aligned with either FliG orientations (CW or CCW) would not give the asymmetrical switch which we systematically observe.

A second hypothetical mechanism could be to assume that the presence of the tags, in proximity of the C ring, prevents the complete conformational switch of the nearest FliG unit. Here the tag, blocking FliG to complete its transition, would reduce, but not necessarily eliminate, the torque in the corresponding conformation. This mechanism relies on the hypothesis of the existence of multiple intermediate functional conformations of FliG.

A third hypothetical possibility could come from the observation that the switching frequency of tagged motors decreases, while the bias is less affected, indicating that the energy barrier between the two conformational states of the FliG protein of the C-ring is increased by the presence of the tag. This could have the effect, in average, to lock a few FliG units in their conformations, depending on the motor bias. The observations could be explained in terms of the conformational spread model, in which it has been shown that locking only a few FliG units can have a strong influence in the switching dynamics of the motor, in terms of bias, maximum speed and residence times [5]. The above speculations remain to be tested.

Fluorescent protein peptide sequences

The sequences of the fluorescent proteins tagged to MotB are listed below. Multiple sequence alignment was performed with Clustal Omega.

```
Dendra2      MNTPGINLIKEDMRVKVHMEGNVNGHAFVIEGEGKGKPYEGTQTANLTVKEGAPLPFSYD
eGFP         MVSKGEELFTGVVPILVELDGDVNGHKFSVSGEGEGDATYGKLTLKFICTT-GKLPVPWP
YPet         -MSKGEELFTGVVPILVELDGDVNGHKFSVSGEGEGDATYGKLTLKLLCTT-GKLPVPWP
               : * :*:.  : : *.::*:**** * :.***:*.   *. * ::  .  . **. :

Dendra2      ILTTAVHYGNRVFTKYPEDI--PDYFKQSFPEGYSWERTMTFEDKGICTIRSDISLEGDC
eGFP         TLVTTLTYGVQCFSRYPDHMKQHDFFKSAMPEGYVQERTIFFKDDGNYKTRAEVKFEGDT
YPet         TLVTTLGYGVQCFARYPDHMKQHDFFKSAMPEGYVQERTIFFKDDGNYKTRAEVKFEGDT
              *.*:: ** : *::**:.:   *:**.::****  ***: *:*.*  . *:::.:***

Dendra2      FFQNVRFKGTNFPPNGPVMQKKTLKWEPSTEKLHV-----RDGLLVGNINMALLLEGGGH
eGFP         LVNRIELKGIDFKEDGNILGHKLE-YNYNSHNVYIMADKQKNGIKV-NFKIRHNIEDGSV
YPet         LVNRIELKGIDFKEDGNILGHKLE-YNYNSHNVYITADKQKNGIKA-NFKIRHNIEDGGV
             :.:.:.:** :*  :* :: :*   :: .:.::::     ::*: . *:::   :*.*.

Dendra2      YLCDFK--TTYKAKKVVQLPDAHFVDHRIEILGNDSDYN-KVKLYEHAVARYSPLP-SQV
eGFP         QLADHYQQNTPIGDGPVLLPDNHYLSTQSALSKDPNEKRDHMVLLEFVTAAGITLGMDEL
YPet         QLADHYQQNTPIGDGPVLLPDNHYLSYQSALFKDPNEKRDHMVLLEFLTAAGITEGMNEL
              *.*.   .*  ..  * *** *::. :  :  : .: . :: * *. .*       .::

Dendra2      W*-
eGFP         YK*
YPet         YK-
             :
```

References

[1] Mark C Leake, Jennifer H Chandler, George H Wadhams, Fan Bai, Richard M Berry, and Judith P Armitage. Stoichiometry and turnover in single, functioning membrane protein complexes. *Nature*, 443(7109):355–358, 2006.

[2] Murray J Tipping, Bradley C Steel, Nicolas J Delalez, Richard M Berry, and Judith P Armitage. Quantification of flagellar motor stator dynamics through in vivo proton-motive force control. *Mol. Microbiol.*, 87(2):338–347, 2013.

[3] Murray J Tipping, Nicolas J Delalez, Ren Lim, Richard M Berry, and Judith P Armitage. Load-dependent assembly of the bacterial flagellar motor. *MBio*, 4(4):e00551–13, 2013.

[4] Kranthi K Mandadapu, Jasmine A Nirody, Richard M Berry, and George Oster. Mechanics of torque generation in the bacterial flagellar motor. *PNAS*, 112(32):E4381–E4389, 2015.

[5] Qi Ma, Yoshiyuki Sowa, Matthew AB Baker, and Fan Bai. Bacterial flagellar motor switch in response to chey-p regulation and motor structural alterations. *Biophys. J.*, 110(6):1411–1420, 2016.
